# Copper induced oxidative stresses, antioxidant responses and phytoremediation potential of Moso bamboo (*Phyllostachys pubescens*)

**DOI:** 10.1038/srep13554

**Published:** 2015-09-04

**Authors:** Junren Chen, Mohammad Shafi, Song Li, Ying Wang, Jiasen Wu, Zhengqian Ye, Danli Peng, Wenbo Yan, Dan Liu

**Affiliations:** 1 Key Laboratory of Soil Contamination Bioremediation of Zhejiang Province, Zhejiang A & F University, Lin’an Zhejiang, 311300, China; 2 The University of Agriculture Peshawar, Pakistan

## Abstract

Moso bamboo is recognized as phytoremediation plant due to production of huge biomass and high tolerance in stressed environment. Hydroponics and pot experiments were conducted to investigate mechanism of copper tolerance and to evaluate copper accumulation capacity of Moso bamboo. In hydroponics experiment there was non significant variation in MDA contents of leaves compared with control. SOD and POD initially indicated enhancing trend with application of 5 μM Cu and then decreased consistently with application of 25 and 100 μM Cu. Application of each additional increment of copper have constantly enhanced proline contents while maximum increase of proline was observed with application of 100 μM copper. In pot experiment chlorophyll and biomass initially showed increasing tendency and decreased gradually with application of each additional increment of Cu. Normal growth of Moso bamboo was observed with application of 100 mg kg^−1^ copper. However, additional application of 300 or 600 mg kg^−1^ copper had significantly inhibited growth of Moso bamboo. The concentration of Cu in Moso bamboo has attained the levels of 340, 60, 23 mg kg^−1^ in roots, stems and leaves respectively. The vacuoles were the main organs which accumulated copper and reduced toxicity of copper as studied by TEM-DEX technology.

Soils an important resource for human survival were seriously contaminated by anthropogenic activities such as mining, municipal sewage and pesticides. In China, more than 16.1% soils are contaminated including 2.1% copper (Cu) contaminated soils. Even though Cu is an essential microelement associated with plant biological processes, but it is also a toxic element due to excessive absorption by plants^1^. Excessive absorption of Cu will disturb cellular processes such as photosynthesis, respiration and cell wall metabolism^2^, disrupt protein structures, inactivate enzymes^3^, inhibit plant growth and endanger survival of plants^4^. Polluted soils may pose serious risks to wildlife and to human health (via food-chain). Therefore, it is of great importance to take measures for remediation of polluted soils.

Phytoremediation is low cost technique with less environmental side effects which utilizes plants to remove heavy metals from environment or to render them harmless^5^,^6^. It is a time-limited process, however, wide use of this technology has been restricted by climatic conditions. Currently at least 25 plant species of Cu hyperaccumulators and large number of Cu accumulators, excluders, and indicators have been identified^7^. Some of the Cu hyperaccumulators or Cu-accumulation species with low biomass capacity are shown in Table 1. Collin *et al.* has confirmed that Bamboos, (*Gigantocloa* sp. “Malay dwarf”) has high tolerance to Cu concentration in hydroponics^8^. Sympodial bamboos (*Phyllostachys fastuosa*) has accumulated 3171 mg kg^−1^ in roots in 4-months hydroponics experiment with application of 100 μM Cu^9^. Previously, few studies have mentioned Moso bamboo (*Phyllostachys pubescens*), even though it has occupied more than 70% of the area of all species of Bamboo in China. We have observed that Moso bamboo has numerous advantages compared to other plants such as quick growth, its biomass production can reach 82 t hm^−2^ of dry weight^10^ and have strong ability to adapt to different environments^11^. Normal growth of Moso bamboo was observed in old Pb/Zn mine area^12^. Moso bamboo has been recognized as potential phytoremediation material for Cu contaminated soils^13^. However, enough information is not available about the extent of tolerance and phytoremediation capacity of Moso bamboo for Cu.

The antixodative defense system in plants plays an important role in reducing toxicity of heavy metals^14^. With excessive Cu stresses, the induction of antioxidative enzymes, including superoxide dismutase (SOD), peroxidase (POD) and catalase (CAT), play an important role in mechanism of reducing Cu toxicity in plants, and plants may secrete some substances to reduce toxicity of Cu^3^,^15^,^16^. The contents of malonaldehyde (MDA) is regarded as membrane destabilization, and proline contents will be accumulated in many species in response to environmental stresses^17^,^18^. Ni *et al.* have employed transmission electron microscopy combined with energy dispersive X-ray analysis (TEM-EDX) technology and revealed that Cu localized in vacuoles of *Elsholtzia splendens* is a mechanism to tolerate Cu toxicity^19^. In case of Moso bamboo, significant studies have not been conducted on mechanism of tolerance in Cu stressed environment.

The characteristics of Moso bamboo such as production of huge biomass and high tolerance to Cu are useful traits for phytoremediation, but effectiveness of plants for decontaminating Cu in soils and antioxidative defense system reactions under Cu stress are unknown. In our experiment, mechanism of antioxidatives in Moso bamboo under Cu stress in hydroponics and soil environment was studied. Transmission electron microscopy-energy dispersive X-ray detector (TEM-EDX) technology was employed to study the organs of plants accumulating Cu.

## Results

### Effects of Cu on biomass and photosynthetic pigments of Moso bamboo in pot experiment

Biomass production of Moso bamboo initially enhanced with increasing Cu levels in the soil. Biomass reached maximum level with application of 50 mg kg^−1^ Cu, and decreased with application of each additional increment of Cu in pot experiment (Table 2). The biomass of roots, stem and leaves were decreased by 66.2%, 54.5% and 84.5% respectively with application of 300 mg kg^−1^ Cu compared with control. The results showed that biomass of leaves were easily affected than roots. Non significant variation was observed in dry weight with application range of 300 and 600 mg kg^−1^ Cu.

Photosynthetic pigments (Chl-a, total Chl-a and Chl-b) exhibited similar response due to exposure of Cu (Table 2). Initially non significant effects on Chl-a, and Chl-a + Chl-b were observed with application of 50 mg kg^−1^ Cu, but increasing trend by 11.7% and 5.8% in Chl-a, and Chl-a + Chl-b respectively was recorded compared with control. Whereas Cu concentration exceeded 100 mg kg^−1^ has inhibited synthesis of pigments and reached minimum value at 600 mg kg^−1^ Cu treatment. The contents of Chl-b were inhibited in all Cu treatment. However, non significant reduction in Chl-b was observed at 50 and 100 mg kg^−1^ compared with significant reduction in Chl-b with application of 300 and 600 mg kg^−1^. The ratio of Chl-a/Chl-b was maintained up to 300 mg kg^−1^ without any significant effect, whereas at 600 mg kg^−1^ the ratio increased significantly compared with control.

### Cu concentration and accumulation at different Cu levels in pot experiment

Cu concentration in shoots (leaves and stem) and roots of Moso bamboo was significantly increased when plants were exposed to different Cu concentrations compared with control (Table 3). Cu accumulation in different tissues of Moso bamboo was highest at 600 mg kg^−1^ in culture medium and increased about 46.3, 13.2 and 4.3 times in roots, stem and leaves respectively compared with control. The translocation factor of Cu has minimum values of 0.18 and 0.07 for stem and leaves respectively. The bioaccumulation factor has revealed minimum values for roots, stem and leaves. Maximum values of TF for stem and leaves were 0.23 and 0.15 respectively in the treatment of 50 mg kg^−1^ Cu. In all Cu levels, the uptake of Cu was high in roots followed by stem and leaves respectively. Non significant variation was observed for leaves accumulating Cu with application of 300 and 600 mg kg^−1^ Cu. In case of bioaccumulation factor, the values greater than 1 were observed in roots with application of 50 and 100 mg kg^−1^ Cu, and reached maximum level in the treatment of 100 mg kg^−1^ Cu.

### Effects of Cu on antioxidation system of leaves and Cu concentration in plants in hydroponics

Table 4 showed antioxidation system of leaves and Cu concentration in different parts of Moso bamboo which differs with application of variable levels of Cu. Non significant differences in contents of MDA were observed in leaves due to increasing Cu levels compared with control. MDA in leaves has slightly increased with high Cu levels and reached maximum level of 0.0269 μM g^−1^ FW from treatment of 100 μM Cu. Application of 100 μM Cu indicated significant increase in proline contents of leaves which showed 133.5% increase compared with control. However, non significant increase of proline from application of 5 and 25 μM Cu stresses was observed. The activity of POD indicated significant increase which revealed maximum value of 38 (U mg^−1^ FW) from treatment of 5 μM Cu compared with 35 and 36 (U mg^−1^ FW) respectively from application of 25 and 100 μM Cu. Application of 5 μM Cu enhanced SOD activity, but further application of Cu at 25 and 100 μM Cu levels indicated downward trend in activity of SOD. The treatment of 100 μM Cu has significantly inhibited the activity of SOD by 3.4% compared with control.

Table 4 revealed that most of Cu was concentrated in roots and fewer was transported to stem and leaves. The Cu concentration was about 20 times in roots, 2.7 times in stem and 2.4 times in leaves respectively with application of 5 μM Cu as compared with control. Supply of excess-Cu increased accumulation of Cu in roots, stem and leaves and all of them showed significant differences compared with control. The Cu concentration in different parts of Moso bamboo can reach 417, 45 and 24 mg kg^−1^ in roots, stem and leaves, respectively with application of 100 μM Cu.

### TEM-EDX

**Figure 1:**
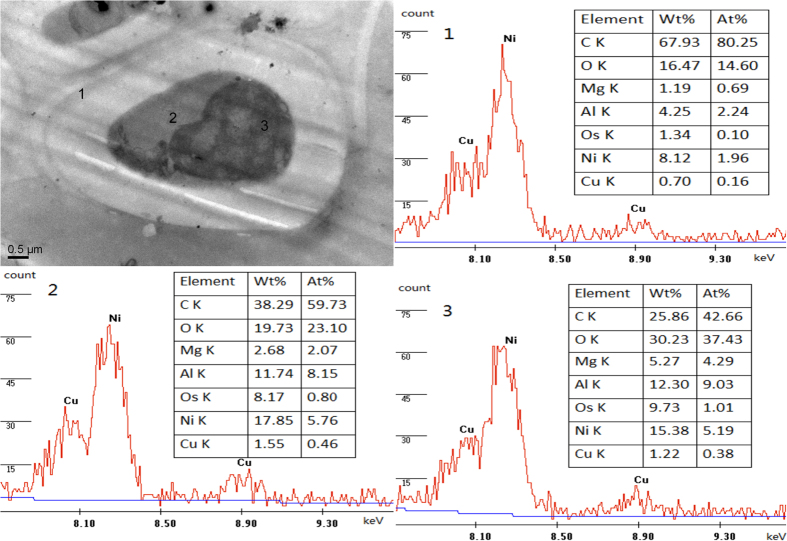
TEM micrographs from stem of Moso bamboo and EDX-specturm of cell wall (1) vacuole (2) and cytoplasm (3).

After application of 100 μM Cu for 10 days, the stem was chosen for TEM-EDX analysis. Cu in cell wall (Fig. 1(1)), vacuole (Fig. 1(2)) and cytoplasm (Fig. 1(3)) were detected. The counts of Cu in Fig. 1 were not high. Element analysis by EDX revealed that Cu in cell was mostly concentrated in vacuole followed by cytoplasm and cell wall respectively (Fig. 1).

## Discussions

### Biomass and photosynthetic pigments of Moso bamboo under Cu stress

Cu is highly toxic for normal plants. Dominant plants discovered in the copper mine area showed more tolerance than normal plants. *Elsholtzia splendens* which was demonstrated as Cu-tolerant species can survive on contaminated soils up to application of 600 mg kg^−1^ Cu^7^. *Sedum sediforme* was able to survive in extreme Cu-toxicity conditions on soils with 5000–16800 mg kg^−1^ Cu in contaminated soil^20^. Moso bamboo was less tolerant than *Elsholtzia splendens*, obviously Moso bamboo will not survive in contaminated soils with more than 300 mg kg^−1^ Cu. Low Cu exposure (<100 mg kg^−1^) does not inhibited plant growth in pot experiment (Table 2). This indicated that Moso bamboo can tolerate low Cu stress and has potential ability to use Moso bamboo as phytoremediation material for low Cu contaminated soil.

Chlorophyll contents is considered to be one of the most important parameter in evaluation of plant stress^21^. The contents of photosynthetic pigments decreased significantly due to high Cu toxicity. Chloroplasts are the main sites for Cu accumulation in higher plants and Cu may affect photosynthesis through its effects at genetic level^22^. Higher Cu has disturbed integrity of thylakoid membranes and has changed their fatty acid composition^23^, and inhibited enzymes associated with chlorophyll biosynthesis^24^. In pot experiment, Cu concentration exceeding 300 mg kg^−1^ has produced significant adverse effect on plants. Which indicated that Moso bamboo could not tolerate more than 300 mg kg^−1^ Cu stress. The ratio of Chl-a/Chl-b means the effectiveness of photosynthesis. Application of 600 mg kg^−1^ Cu resulted in Chl-a/Chl-b ratio of 8.31. It was an abnormal value which has disturbed the progress of synthesis of Chl-a and Chl-b. It is revealed that Chl-a contents have reached maximum level 1.82 (mg g^−1^) with application of 50 mg kg^−1^ Cu compared with 1.63 (mg g^−1^) from control. Application of higher Cu levels has caused consistent significant reduction in Chl-a. Non significant reduction was observed for Chl-b at low Cu exposure (<100 mg kg^−1^). Low Cu levels has not affected plant growth because Cu is one of the essential element and plays an important role in plant growth.

### Cu accumulation in plant tissues

The translocation factor (TF) and bioaccumulation factor (BCF) are important in screening hyperaccumulators for phytoremediation of heavy metals. Screening of hyperaccumulators depend on BCF and TF values (both of them are greater than 1) for evaluation and selection of plants for phytoremediation^25^. The TF is the capacity of plants to transfer metals from roots to shoots and BCF express the ability of plants to accumulate metals from soils to tissues^26^. Another requirement for criteria of plants whether it is Cu hyperaccumulator species or not is Cu accumulation in shoots. Cu accumulation in shoots should be greater than 1000 mg kg^−1^ dry weight when grown on metals rich soils^27^. Obviously, Moso bamboo is not hyperaccumulator for Cu. Most of Cu absorbed from soil was accumulated in roots and all BAF and TF values were less than 1 except BAF of roots at 100 mg kg^−1^ Cu treatments. Our values of TF for copper were lower than *Cyperus eculentus*, *Gentiana pennelliana*, and *Stenotaphrum secundatum* with values of 2.8, 0.56, and 0.77, respectively^28^. Compared to *Elsholtzia splendens*, with TF value of 0.006 at NH_4_OAc extractable soil Cu level of 114 mg kg^−1^, Moso bamboo was more efficient to transport Cu to shoots^7^.

Maximum Cu concentration in Moso bamboo was 340, 60 and 23 mg kg^−1^ for roots, stem and leaves with applicaton of 600 mg kg^−1^ Cu, which almost expired the plants of Moso bamboo. Average concentration of Cu in *Commelina communis Linn*. and *Rumex acetosa Linn*. have reached 157 and 601 mg kg^−1^ which were dominant species in Cu mining soils^29^. The *Hirschfeldia incana* could survive the soil Cu concentration up to 4930 mg kg^−1^ in roots and Cu concentration of 355 mg kg^−1^ in shoots without any toxicity^20^. All of these species showed stronger ability to absorb Cu from soils to shoots than Moso bamboo. Application of 300 mg kg^−1^ Cu has significantly affected growth of Moso bamboo as biomass decreased sharply as compared with 100 mg kg^−1^ Cu treatment (Table 2). This may due to high exchangeable or available Cu concentration in the soil after application of more than 300 mg kg^−1^ Cu in soil. The extractable Cu concentration in contaminated soil is lower than in pot experiment with in few days after application of soluble Cu in soil. Near the mine area where dominant plant is Moso bamboo, the total Cu concentration can reach 360 mg kg^−1^ and NH_4_OAc extracted Cu was 14 mg kg^−1^^12^. The value of NH_4_OAc extracted Cu in 400 mg kg^−1^ Cu treatment in pot experiment could reach 40 mg kg^−1^^7^. The seedling of Moso bamboo is quite different from mature plant.

Removal of Cu from contaminated soils by *Elsholtzia splendens* was remarkable and *Elsholtzia splendens* could remove 1.7 kg Cu ha^−1^ from soil^7^. The highest potential copper phytoremediation by *Archis pintoi* was in contaminated soil with copper removal of 2.5 kg ha^−1^^20^. Moso bamboo has produced 82 t ha^−1^ dry mass whereas more than 57% of dry matter was from stem and 28% from roots^10^. Growth of Moso bamboo was normal with application of 100 mg kg^−1^ Cu however, Cu concentration in roots, stem and leaves has reached 178, 33 and 16 mg kg^−1^ respectively. Accordingly Moso bamboo could remove Cu 9.3 kg ha^−1^ from soils. Obviously Moso bamboo as phytoremediation material has removed high quantity of Cu from soil compared with *Elsholtzia splendens* and *Archis pintoi*. Further field experiments are suggested in future before Moso bamboo is widely used as phytoremediation material for Cu contaminated soils.

### Effects of Cu on antioxidation system of leaves and Cu contents of plants in hydroponics

Absorption and accumulation of Cu in roots, stems, leaves and seeds was enhanced with increasing exogenous Cu levels. It was related to plant species and incubation time in hydroponics. Compared with other Cu tolerant plants, such as *Elsholtzia splendens* which has great potential for phytoremediation^30^, Cu concentration can reach 3417 mg kg^−1^ in its shoot. In our results, Cu concentration in leaves, stem and roots have reached 24, 45 and 417 mg kg^−1^ respectively with application of 100 μM Cu. Cu concentrations in tissues of Moso bamboo was weak for phytoremediation, it may due to short duration growth of Moso bamboo in hydroponics. Chen *et al.* reported that Cu concentrations have reached 809 and 91 mg kg^−1^ in roots and shoot of Moso bamboo after application of Cu for 30 days in hydroponics, which may due to exposure of Moso bamboo to Cu for long period of time^13^.

The cell membranes of plants are considered as primary sites of injury due to heavy metals and membrane destabilization was frequently attributed to lipid peroxidation^17^. Large number of active oxygen free radicals in plant tissues under stress will cause cell membrane lipid peroxidation, which will damage normal structure and function of membrane. MDA is an oxidized product of membrane lipids, which is commonly considered general indicator of lipid peroxidation as well as stress level^1^. In this study, MDA contents of treated plants under Cu stress showed non significant difference than control, but the contents of MDA treated with 100 μM Cu were highest in all treatments. It indicated that excess Cu didn’t increase lipid peroxidation and induced oxidative stress in leaves of Cu-stressed plants. That may due to large amount of Cu was accumulated in roots and fewer transported to shoots or leaves. If treated with more than 100 μM Cu it may induce lipid peroxidation in leaves. It was observed in the leaves of *Matricaria chamomilla* that content of MDA showed non-significant difference in all copper treatments^31^. The reactive oxygen species were produced by plants under stresses of heavy metals and it rapidly attacked all types of biomolecules such as nucleic acids, proteins, lipids and amino acids^32^. It dismutated to H_2_O and finally to O_2_ by enzymes of SOD and POD, which are essential component of plant antioxidation system. Our results showed increase in SOD and POD activity at 5 μM Cu treatment however, it decreased at 25 and 100 μM Cu treatments. The results indicated that Moso bamboo can increase the activity of SOD and POD to protect it from oxidative damage induced by Cu toxicity, but high Cu stress has caused large amount of reactive oxygen species which inhibited enzymes activity. Treatments with high Cu levels indicated non-significant difference in both POD and SOD compared with control which may be due to low Cu concentration in leaves. In leaves of *Oryza sativa* L. the activities of SOD were lower than roots, while Cu concentration of roots was 10 folds higher than leaves^3^.

Plants have secreted proline as osmoregulatory solute to reduce toxic effect when exposed to wide variety of environmental stresses which provided stress tolerance^16^. The generation of proline is one of the vital responses of plants under Cu toxicity, which is possibly associated with protection of plant cells against oxidative damage and with signal transduction^15^. In this study the contents of proline increased with enhancing Cu levels, and non-significant variation was observed for 5 and 25 μM Cu treatments compared with control. However, further increase of proline was observed with application of 100 μM Cu. It indicated that Cu in leaves has disturbed the balance of osmoregulatory solutes, but had no real impact for plants. The increasing contents of proline with application of 100 μM Cu may be due to the fact that concentration of Cu has broken osmoregulatory solutes and has stimulated proline synthesis. Mostofa and Fujita reported that application of Cu has doubled the contents of proline and it can be increased further more than 6 times in *Oryza sativa* L^3^.

### Cu localization

Cell wall is regarded as first protective barrier to stop entry of heavy metals in the cell. Nishizono *et al.* reported that about 70%–90% accumulated Cu was concentrated in cell wall of *Athyrium yokoscense*^33^. Using TEM-EDX technology, it was reported that mostly Cu was accumulated in vacuoles and cytoplasm^34^. The big dark particles deposited in cell were Cu or not, which still need to be investigated in future research studies. Sequestration of metal ions and metal-chelate complexes is an important aspect of metal ions detoxification in hyperaccumulator plants^35^. Vacuoles and cytoplasm contain plenty of organic acid, enzyme, protein and lipid that can chelate metals to reduce toxicity which may be the reason why Cu was mostly concentrated in vacuoles and cytoplasm. Our results are in agreement with Ni *et al.*, they reported that vacuoles have played an important role in Cu tolerance of Moso bamboo and it was the main organ which has accumulated Cu^36^.

## Conclusions

Ideal plants for phytoremediation should possess an extensive root system with maximum production of biomass in presence of high concentration of heavy metals. On the whole, physiological/biochemical characterization of the responses of Moso bamboo to varying Cu concentrations can be of great help in elaborating the innovative plant-based remediation technologies for heavy metal contaminated sites. All considered parameters indicated that Moso bamboo can tolerate highest concentration of 300 mg kg^−1^ of Cu. The results of this study revealed useful information regarding established ability of Moso bamboo to transport, and to delocalize Cu in aboveground biomass and phytoremediation strategies for Cu contaminated soils. The uptake of Cu by Moso bamboo in Cu polluted soils with subsequent utilization of Cu was calculated. Highest Cu concentration has caused ultrastructural alteration in stems cells. Inspite of several shortcomings such as slow growth, low biomass and regional types in the already identified Cu-hyperaccumulators, the investigation of Moso bamboo will fill a gap of known phytoremediation plants. It is concluded that Moso bamboo could be regarded as a candidate species for the phytoremediation of Cu contaminated soil.

## Methods

### Hydroponics experiment

Plants were cultured under glasshouse environment with natural light, day/night temperature of 25/30 °C, and day/night humidity of 70/90%. Seeds of *Phyllostachys pubescens* collected from Guilin of Guangxi Province, China were sown in substrate containing perlite and vermiculite (3:1 v/v) moistened with distilled water. The nutrients solution was applied until seedlings had two leaves pairs. The composition of nutrients solution was as follows (mg L^−1^): 57.1 NH_4_NO_3_, 25.2 NaH_2_PO_4_·2H_2_O, 44.6 K_2_SO_4_, 55.4 CaCl_2_, 202.5 MgSO_4_·7H_2_O, 4.7 Na_2_EDTA, 3.5 FeSO_4_·7H_2_O, 0.8 MnSO_4_·H_2_O, 0.05 (NH_4_)_6_MO_7_O_24_·4H_2_O, 0.6 H_3_BO_3_, 0.02 ZnSO_4_·7H_2_O, 0.02 CuSO_4_·5H_2_O, 7.4 Citric acid (monohydrate), and 0.0625 mL H_2_SO_4_. After three weeks, seedlings of uniform size were selected and transferred to plastic pots containing nutrients solution. All seedlings of Moso bamboo were exposed to uniform growth environment. Ten plants were chosen randomly in each pot as one replicate and arranged randomly with each treatment in triplicate. Cu was applied as CuSO_4_·5H_2_O at (1) 0 μM (control), (2) 5 μM, (3) 25 μM, (4) 100 μM. The pH value of the nutrients solution was adjusted to 5.8 with 0.1 M NaOH or 0.1 M HCl. The nutrients solution was continuously aerated and renewed after every 5d.

### Plants harvest

Cu treated plants were harvested after 15 days of growth. At the time of harvest, the intact plants were washed with distilled water, and immersed in 20 mM Na_2_EDTA for 15–20 min to remove Cu adhered to roots surface. Plants were washed thrice with distilled water and finally with de-ionized water. All plants were divided in roots, stem and leaves for further analysis.

### Elemental analysis

Roots, stem and leaves were dried at 70 °C for approximately 72 h, and dry weight was recorded soon. The oven-dried plant parts were grounded in stainless steel mill, and passed through 0.1 mm nylon sieve for Cu analysis. Approximately 0.3 g of plant samples were digested in HNO_3_/HClO_4_ solution. The digested solution was washed in 50 ml flasks and volume was made using de-ionized water. The supernatant was filtered through 0.45 μm filter paper, acidified with 15% HNO_3_ and finally analyzed for Cu concentration by ICP-MS (Agilent 7500a).

### Determination of contents of lipid peroxidation

Fresh leaves samples of 0.5 g from Cu treated plants were grounded with liquid nitrogen and dissolved in 10 ml of cold 50 mM potassium phosphate buffer (pH 7.8) contaning 0.2 mM EDTA and 2% (w/v) polyvinylpyrrolidone (PVP). The homogenate was centrifuged for 20 min at 10000 rpm at 4 °C and supernatant was used for analysis. Lipid peroxidation was measured by amount of MDA, a product of unsaturated fatty acid peroxidation. MDA concentration was determined according to Liu *et al.*^37^.

### Determination of proline contents

Proline (Pro) contents were determined according to method of Saradhi^38^ using acidninhydrin solution. Fresh tissues of leaves (about 0.5 g) were homogenated with 5 ml 3% sulfosalicylic acid and the homogenate was centrifuged at 4000 g for 20 min. 2 ml aliguot of the supernatant was transferred in test tube, 2 ml acetic acid and 2 ml acid ninhydrin were added, and mixture was boiled for 30 min. The reaction was terminated in an ice bath. The reaction mixture was extracted with 4 ml toluene, mixed thoroughly by vortex. The optical density of the upper toluene phase was determined at wavelength of 520 nm.

### Assay of antioxidant enzymes

The supernatant for proline determination was used for determination of superoxide dismutase (SOD) and peroxidase (POD) activity.Superoxide dismutase activity was determined by photochemical method as described by Giannopolitis and Ries^39^. One unit of SOD activity was defined as the amount of enzyme required to cause 50% inhibition of the rate of nitrobluetetrazolium (NBT) reduction as measured at 560 nm. The activity of POD was determined as described by Meng *et al.*^1^ and 0.1 ml supernatant was used for analysis. Activity was measured by an increase in absorbance at 470 nm due to guaiacol oxidation. The results are persented as ΔOD_470nm_ (0.01) per min per mg fresh weight.

### Transmission electron microscopy (TEM) and energy dispersive X-ray detector (EDX) analysis

Plants treated with 100 μM Cu were selected for the TEM and EDX studies. Small sections of 1–3 mm length of mid stem were used for TEM studies. Sections were fixed in 4% glutaraldehyde (v/v) in 0.1 M PBS (sodium phosphate buffer, pH 7.0) for 6–8 h and post-fixed in 1% OsO_4_ (Osmium (VIII) oxide) for 1 h and 0.1 M PBS (pH 7.0) for 1–2 h. Dehydration was carried out using graded series of ethanol (50%, 70%, 80%, 90%, 95% and 100%) for about 15 to 20 minutes at each step. The samples were filtered and embedded in Spurr’s resin. Ultra-thin sections (80 nm) were prepared and mounted on Ni-grids for viewing in transmission electron microscope (JEOL TEM-1200EX) at an accelerating voltage of 60.0 kV then used for EDX (EDAX Genesis XM 2) analysis.

### Pot experiment

Uncontaminated farm soil was procured from Zhejiang A & F University, China. The soils were air-dried and sieved through 5 mm sieve. The main agrochemical properties of the tested soil are: pH 5.52 (water/soil = 2.5:1), organic matter 64.96 g kg^−1^, alkali-hydrolyzale N 225.1 mg kg^−1^, rapid available P 9.0 mg kg^−1^, rapid available K 80.64 mg kg^−1^, and total Cu 26.55 mg kg^−1^^40^. The soils were artificially contaminated with Cu at concentration of 0, 50, 100, 300, 600 mg kg^−1^, respectively. Source of Cu was CuSO_4_**•**5H_2_O. After adding heavy metals, the soils were equilibrated for 6 months, undergoing 7 cycles of saturation with distilled water and air-drying.

The seedlings of Moso bamboo were the same as used for hydroponics experiment. Uniform seedlings were selected after two weeks growth in nutrients solution and each pot contains 1 kg soils with three plants. The soil moisture content was maintained at 60% (w/w) of water-holding capacity by addling de-ionized water after every 2 days. Each pot as one replicate was arranged randomly with each treatment in triplicate. Plants were grown under glasshouse environment with natural light, day/night temperature of 25/30 °C, and day/night humidity of 70/90%.

### Plants harvest

Plants were harvested after 2months. After harvest, the intact plants were washed with distilled water, and immersed in 20 mM Na_2_EDTA for 15–20 min to remove Cu adhered to root surface. Plants were washed thrice with distilled water and finally with de-ionized water, All plants were divided in roots, stem and leaves for further analysis. Element analysis was the same as carried out for previous hydroponics experiment. Bioaccumulation factor (BAF) = [Cu contents in tissues]/[Cu contents in soil]; translocation factor (TF) = [Cu contents in stem]/[Cu contents in roots] and [Cu contents in leaves]/[Cu contents in roots].

### Chlorophyll contents

The method of chlorophyll contents was according to Liu *et al.*^41^. At the time of harvest, fresh leaves of seedlings were collected for determination of chlorophyll contents. Leaves were cut in small pieces, and 0.2 g of the samples was placed in glass tubes. Then 10 ml of 80% acetone was added in samples and kept for 24 h for complete extraction. Chlorophyll contents were determined spectrophotometrically using visible wavelengths of 663 and 645 for chlorophyll a and chlorophyll b.

### Statistical analysis

Statistical analysis was performed with statistical package SPSS (v. 21.0). All values reported are means of three independent replicates. Data were tested at significant levels of *P* < 0.05 by one-way ANOVA (LSD). Graphical work was carried out using Sigma plot software v.12.5.

## Additional Information

**How to cite this article**: Chen, J. *et al.* Copper induced oxidative stresses, antioxidant responses and phytoremediation potential of Moso bamboo (*Phyllostachys pubescens*). *Sci. Rep.* **5**, 13554; doi: 10.1038/srep13554 (2015).

## Figures and Tables

**Table 1:** Cu concentration in tissues of some Cu-tolerant and Cu-hyperaccumulators.

**Species**	**Concentration (mg kg**^**−1**^)					Biomass(dry weight)	**Reference**
	**Soil**	**Shoot**	**Leaves**	**Stem**	**Roots**		
*Petridium revolutum*	2432	–	201	–	346	27 g plant^−1^	42
*Rumex acetosa*	11138	–	596	80	122	7 t ha^−1^	29
*Commelina communis*	13077	–	512	584	3768	7 t ha^−1^	29
*Elsholtzia haichowensis*	14170	–	154	79	987	8 t ha^−1^	29
*Elsholtzia splendens*	1200	9.7	–	–	1751	4.5 t ha^−1^	7,43
*Arachis pintoi*	142	52	–	–	475	3 t ha^−1^	44
*Ricinus communis*	1927	–	551	394	2346	2.5 t ha^−1^	45
*Polygonum microcephalum*	1494	–	133	110	491	15 g plant^−1^	46
*Rumex hastatus*	2105	–	45	42	33	53 g plant^−1^	46

**Table 2:** Biomass and chlorophyll of Moso bamboo as affected by copper stress in pots for two months.

Cuconcentration(mg kg^−1^)	Roots DM(g plant^−1^)	Stem DM(g plant^−1^)	Leaves DM(g plant^−1^)	Chl-a(mg g^−1^)	Chl-b(mg g^−1^)	Chl-a + Chl-b(mg g^−1^)	**Chl-a/Chl-b**
0	0.83 ± 0.08b	0.36 ± 0.03a	2.20 ± 0.29ab	1.63 ± 0.03ab	0.45 ± 0.03a	2.07 ± 0.06a	3.64 ± 0.15a
50	1.00 ± 0.05a	0.37 ± 0.03a	2.63 ± 0.28a	1.82 ± 0.09a	0.38 ± 0.07a	2.19 ± 0.11a	4.91 ± 0.97a
100	0.77 ± 0.08b	0.33 ± 0.06a	2.13 ± 0.34b	1.54 ± 0.14b	0.43 ± 0.06a	1.97 ± 0.18a	3.56 ± 0.40a
300	0.26 ± 0.04c	0.15 ± 0.03b	0.33 ± 0.03c	0.76 ± 0.16c	0.20 ± 0.04b	0.96 ± 0.19b	3.75 ± 0.38a
600	0.16 ± 0.03c	0.10 ± 0.01b	0.17 ± 0.09c	0.35 ± 0.04d	0.04 ± 0.01c	0.40 ± 0.06c	8.31 ± 1.9b

Data points and error bars represent mean ± S.D. of three replicates (n = 3). Different letters indicate significant difference (*P* < 0.05).

**Table 3:** Copper concentrations in roots, stems, leaves, bioaccumulation factor (BAF) and translocation factor (TF) of copper uptake in Moso bamboo for 2 months in pots.

Cu conc.(mg kg^−1^)	Roots(mg kg^−1^)	Stems(mg kg^−1^)	Leaves(mg kg^−1^)	**BAF**			**TF**	
				**Root**	**Stem**	**Leaves**	**Stem**	**Leaves**
0	7 ± 2.8d	5 ± 0.5e	5.4 ± 0.2d	–	–	–	–	–
50	60 ± 6c	14 ± 1.5d	9 ± 1c	0.78	0.18	0.12	0.23	0.15
100	178 ± 28b	33 ± 3c	16 ± 1.4b	1.41	0.26	0.13	0.18	0.09
300	207 ± 32b	46 ± 3b	21 ± 3a	0.63	0.14	0.06	0.22	0.10
600	340 ± 21a	60 ± 7a	23 ± 4a	0.54	0.04	0.04	0.18	0.07

Data points and error bars represent mean ± S.D. of three replicates (n = 3). Different letters indicate significant difference (*P* < 0.05).

**Table 4:** Activity of SOD, POD, MDA contents and proline of Moso bamboo for 15 days copper stress in hydroponics.

Cuconc.(μM)	Roots(mg kg^−1^)	Stem(mg kg^−1^)	Leaves(mg kg^−1^)	MDA contents(μM g^−1^ FW)	Proline contents(μg g^−1^ FW)	POD activity(U mg^−1^ FW)	SOD activity(U g^−1^ FW)
0	8.4 ± 0.8d	4.4 ± 1.2d	3.5 ± 0.6c	0.0224 ± 0.0021a	14.4 ± 0.7b	38 ± 2ab	161 ± 12ab
5	165 ± 23c	12 ± 2.9c	8.3 ± 2.5bc	0.0236 ± 0.003a	16.0 ± 2.2b	43 ± 4a	192 ± 12a
25	344 ± 29b	29 ± 6b	14 ± 2.3b	0.233 ± 0.0023a	16.3 ± 3.5b	35 ± 5b	168 ± 23ab
100	417 ± 57a	45 ± 10a	24 ± 5a	0.0269 ± 0.0032a	33.6 ± 8.1a	36 ± 2b	156 ± 22b

Data points and error bars represent mean ± S.D. of three replicates (n = 3). Different letters indicate significant difference (*P* < 0.05).
